# Targeting CD22 for B-cell hematologic malignancies

**DOI:** 10.1186/s40164-023-00454-7

**Published:** 2023-10-11

**Authors:** Jia Xu, Wenjing Luo, Chenggong Li, Heng Mei

**Affiliations:** 1 Hubei Clinical Medical Center of Cell Therapy for Neoplastic Disease, Wuhan, 430022 China; 2 Institute of Hematology, Union Hospital, Tongji Medical College, Huazhong University of Science and Technology, 1277 Jiefang Avenue, Wuhan, 430022 Hubei China

**Keywords:** CD22, CD22 CAR-T cell therapy, CD22 antibody–drug conjugate, Dual-targeting CAR-T cell, Combination therapies

## Abstract

CD19-targeted chimeric receptor antigen (CAR)-T cell therapy has shown remarkable clinical efficacy in the treatment of relapsed or refractory (R/R) B-cell malignancies. However, 30%–60% of patients eventually relapsed, with the CD19-negative relapse being an important hurdle to sustained remission. CD22 expression is independent of CD19 expression in malignant B cells. Consequently, CD22 is a potential alternative target for CD19 CAR-T cell-resistant patients. CD22-targeted therapies, mainly including the antibody–drug conjugates (ADCs) and CAR-T cells, have come into wide clinical use with acceptable toxicities and promising efficacy. In this review, we explore the molecular and physiological characteristics of CD22, development of CD22 ADCs and CAR-T cells, and the available clinical data on CD22 ADCs and CAR-T cell therapies. Furthermore, we propose some perspectives for overcoming tumor escape and enhancing the efficacy of CD22-targeted therapies.

## Background

Chimeric antigen receptor (CAR)-T cell therapy has attracted much attention as a cellular immunotherapy. Although CD19 CAR-T cell therapy has achieved promising efficacy in clinical settings [1–5], 30%–60% of patients eventually relapsed with a poor prognosis [6–14]. One mechanism of relapse is the downregulation or loss of CD19 on the tumor cell surface [15–17]. In B-cell acute lymphoblastic leukemia (B-ALL), CD19-negative relapse accounts for up to 83% of relapse cases [6–11, 18]. In B-cell non-Hodgkin lymphoma (NHL), antigen loss also occurs in one–third of patients experiencing treatment failure after CD19 CAR-T cell therapy [15, 19].

CD22 is restrictively expressed in both normal and malignant B cells, which makes it a potential alternative target to CD19 [20, 21]. CD22 has been identified in the blasts of > 90% of B-ALL cases [22, 23]. Studies on the applicability of targeting CD22 have mainly focused on antibody–drug conjugate (ADC) and CAR-T cell therapy. Two CD22 ADCs, inotuzumab ozogamicin and moxetumomab pasudotox-tdfk, have been approved by the Food and Drug Administration for the treatment of relapsed or refractory (R/R) B-ALL and hairy-cell leukemia, respectively. While inotuzumab ozogamicin is increasingly used in therapeutic settings, moxetumomab pasudotox-tdfk was withdrawn from the market due to the low clinical uptake and complexity of the drug use. CD22 CAR-T cell therapies prove to be effective in treating R/R B-ALL [20, 21, 24–31]. However, treatment failure is inevitable in CD22-targeted immunotherapies, further narrowing down the avaliable treatment options. Comprehensive knowledge of CD22 molecule will help us to understand the mechanisms of resistance and propose corresponding strategies. This review focuses on the development, the available clinical data and the strategies to improve the efficacy of CD22-targeted therapies, with the aim of providing a perspective on future directions in CD22-targeted immunotherapies.

## CD22 structure, function, and expression

**Fig. 1:**
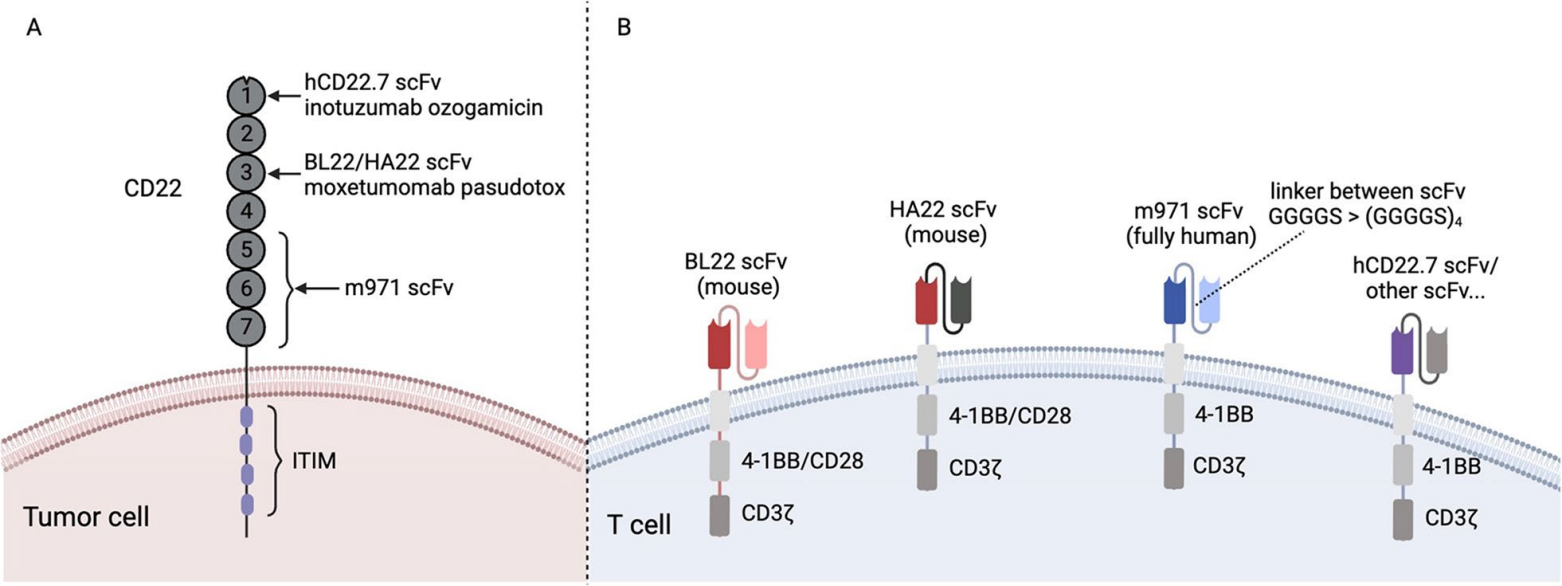
CD22 and CD22 CAR structure. A CD22 molecule and the recognized domain of different CD22 scFv and ADCs. B CD22 CAR structures in preclinical and clinical use. ITIM immunoreceptor tyrosine-based inhibitory motif. The image was created using BioRender (Biorender, Toronto, ON, Canada)

CD22, also known as sialic acid-binding Ig-like lectin 2, belongs to the siglec family and immunoglobulin superfamily. It consists of seven extracellular IgG-like domains and a 141-amino acid-long cytoplasmic tail [32, 33] (Fig. 1A). CD22 can bind to α 2,6-linked sialic acid residues of surface molecules (such as CD22 itself, CD45 and IgM) on B cells in a “cis” configuration. It can also bind to ligands on other cells as an adhesion molecule in a “trans” configuration [34, 35]. Cis-ligation negatively tunes B-cell receptor signaling mainly in a SH2 domain-containing tyrosine phosphatases 1-dependent manner. Trans-ligation modulates the migration and B-cell receptor signaling threshold of B cells [33, 34]. CD22 is restrictively expressed in the B-cell lineage, particularly in malignant B cells [23, 34, 36–40]. As an endocytic receptor, ligation of CD22 with its ligands triggers rapid internalization, which enables the application of CD22 ADCs [41].

## CD22 ADC structures and preclinical results

ADC is composed of an antibody, a chemical linker, and a covalently attached cytotoxic agent (i.e., the payload). The ADC undergoes endocytosis after the antibody binds to the specific antigen on the tumor cell surface, and then releases the payload from the lysosomes. Choosing an optimal antibody is the first step in developing CD22 ADCs. The payload also plays a pivotal role in triggering the crosslinking or breakage of DNA, or inhibiting the tubulin activity, thus leading to cell cycle arrest and tumor cell apoptosis. The linker determines the stability of ADCs and also controls the release of payloads; it is designed to prevent unnecessary clustering of ADCs, which impairs the anti-tumor activity [42, 43]. The structures of CD22 ADCs/recombinant immunotoxins are summarized in Table 1.

**Table 1:** CD22 ADC/recombinant immunotoxin structures

ADC/recombinant immunotoxin	Anti-CD22 antibody	Cytotoxic payload	Linker	Disease
BL22	RFB4 dsFv	Pseudomonas exotoxin A (PE38)	mc-VC-PABC (enzyme cleavable)	R/R HCL
Moxetumomab pasudotox/HA22	RFB4 dsFv (SSY-THW)	Pseudomonas exotoxin A (PE38)	mc-VC-PABC (enzyme cleavable)	R/R B-cell HCL
Pinatuzumab vedotin	Hu10F4 antibody	Monomethyl auristatin E	mc-VC-PABC (enzyme cleavable)	R/R B-cell NHL
Anti-CD22-NMS249	Hu10F4 antibody	PNU-159682	mc-VC-PABC (enzyme cleavable)	R/R B-cell NHL
Anti-CD22-(LC:K149C)-SN36248	Hu10F4 antibody	SN36248 × 2	maleimide linker (uncleavable)	B-cell NHL
Inotuzumab ozogamicin	G544 antibody	Calicheamicin (Calich-DMH)	hydrazone (acid-labile linker)	R/R B-ALL

*dsFv* disulfide-stabilized Fv fragment, *R/R* relapsed or refractory, *HCL* hairy cell leukemia, *NHL* non-Hodgkin lymphoma, *ALL* acute lymphoplastic leukemia

BL22 contains a disulfide-stabilized anti-CD22 fragment variable derived from a murine RFB4 antibody and a 38 kDa truncated form of pseudomonas exotoxin A [44]. HA22 is a refined form of BL22 that shows a higher affinity to CD22. In HA22, SSY residues in the hot spot region of complementarity-determining region 3 of heavy chain of BL22 were mutated to THW residues [45, 46]. HA22 showed improved in vivo and in vitro anti-tumor activity compared to BL22 [45]. Unlike moxetumomab pasudotox deriving from HA22, inotuzumab ozogamicin comprises the humanized anti-CD22 monoclonal IgG4 antibody G544 and a chemically linked DNA-damaging payload Calich-DMH. It recognizes the IgG-like domain 1 of CD22 and exerts a potent cytotoxic effect on tumor cells, leading to an obvious tumor mass regression in two lymphoma-xenograft bearing models [47, 48]. Pinatuzumab vedotin incorporates the humanized anti-CD22 monoclonal IgG1 antibody Hu10F4 and a microtubule inhibitor monomethyl auristatin E. Pinatuzumab vedotin demonstrated better anti-tumor activity than the standard rituximab plus CHOP regimen in a xenograft model bearing Ramos cells and also showed anti-lymphoma effects in vitro [49]. CD22-NMS249, with the same antigen-binding region of pinatuzumab vedotin and a more potent anthracycline analogue PNU-159682, displayed better cytotoxicity than pinatuzumab vedotin [50]. Anti-CD22-(LC:K149C)-SN36248 uses the same antibody of pinatuzumab vedotin and two SN36248 molecules as payload. The K149C conjugation site in the light chain promotes the in vivo conjugation stability of the ADC. SN36248 is a non-cleavable linker drug compound with a seco-CBI homodimer tethered to a maleimide linker. Anti-CD22-(LC:K149C)-SN36248 yielded a longer response duration than pinatuzumab vedotin and showed anti-lymphoma effects in several mouse models, including two resistant to pinatuzumab vedotin (Raji and WUS-DLCL2) [51].

## Efficacy and safety of CD22 ADCs

The available clinical results [52–62] for CD22 ADCs in treating B-ALL and B-cell NHL are summarized in Table 2. Inotuzumab ozogamicin was evaluated in 90 patients with R/R B-ALL in a phase 2 study [54] and showed a complete response (CR) rate of 58% and a median duration of remission of 7 months. Seven patients (6.7%) experienced veno‐occlusive disease (VOD)/sinusoidal obstruction syndrome (SOS), among whom six developed VOD/SOS after transplantation. A two-arm, randomized phase 3 study [55, 56] compared the efficacy of inotuzumab ozogamicin (n = 164) and standard chemotherapy (n = 143) in adult patients with R/R B-ALL. The CR rate was also higher in the inotuzumab ozogamicin group (74% vs 31%). The median progression-free survival (PFS) and overall survival (OS) in the inotuzumab ozogamicin group were 5 and 7.7 months, respectively. Notably, the incidence of VOD/SOS was higher in the inotuzumab ozogamicin group (14% vs 2%). A phase 2 study assessed inotuzumab ozogamicin in 48 pediatric and adolescent patients with R/R B-ALL, among whom 10 (20.8%) underwent prior CD19 CAR-T cell therapy, 1 (2.1%) received CD22 CAR-T cell infusion, and 14 (29.2%) were treated with blinatumomab (a CD3/CD19 bispecific T cell engager). This study reported a CR rate of 58.3%, and minimal residual disease-negativity (< 0.01%) was achieved in 66.7% of patients. Twenty-one patients then proceeded to hematopoietic stem cell transplantation (HSCT) after ADC treatment, and six patients (28.6%) developed SOS after HSCT.

**Table 2:** Results of clinical trials of CD22 ADCs in B-ALL, B-cell NHL and CLL (single-agent)

Clinical trial information	Agent	Institution	Disease and patients (single agent cohort)	Prior CD19-targeted therapy	ORR(≥ CR, best response)	Veno-occlusive disease	Median PFS	Median OS
Phase 1 NCT00717925 [52]	Inotuzumab ozogamicin	Nagoya Daini Red Cross Hospital	R/R FL 13 pts	Rituximab 100%	85% (54%)	-	-	-
Phase 1 [53]	Inotuzumab ozogamicin	Multicenter	R/R B-cell NHL 79 pts	-	FL (MTD): 68% (-) DLBCL (MTD): 15% (-)	1.30%	FL: 317 days DLBCL: 49 days	FL: not reached DLBCL: 193 days
Phase 2 NCT01134575 [54]	Inotuzumab ozogamicin	MD Anderson Cancer Center	R/R B-ALL 90 pts	-	58% (58%)	6.7%	mDOR 7 mos	6.2 mos
Phase 3, 2-arm NCT01564784 (INO-VATE) [55] [56]	Inotuzumab ozogamicin	Multicenter	R/R B-ALL 164 pts	-	- (74%)	14%	5 mos	7.7 mos
Phase 2 NCT00868608 [57]	Inotuzumab ozogamicin	Multicenter	Refractory indolent B-NHL 81 pts	-	67% (31%)	-	12.7 mos	not reached
Phase 1/2 NCT01363297 [58]	Inotuzumab ozogamicin	Multicenter	R/R B-ALL 72 pts	-	-(68%)	5.6%	3.9 mos	7.4 mos
Phase 1 EUDRA-CT 2016–000227-71 [59]	Inotuzumab ozogamicin	Multicenter	R/R B-ALL 25 pediatric pts	Blinatumomab 24% CAR-T 4%	80% (60%)	8%	-	DL1: 7.2 mos DL2: not reached
Phase 2 EUDRA-CT 2016–000227-71 [60]	Inotuzumab ozogamicin	Multicenter	R/R B-ALL 28 pediatric pts	Blinatumomab 25%	82% (82%) in 27 evaluable pts	25%	1-year EFS 36.7%	1-year OS 55.1%
Phase 2 NCT02981628 [61]	Inotuzumab ozogamicin	Multicenter	R/R B-ALL 48 pts	CAR-T 23% Blinatumomab 29%	65% (58%)	13%	2-year EFS 28.6%	2-year OS 36%
Phase 1 NCT01209130 [62]	Pinatuzumab vedotin	Multicenter	R/R DLBCL 25 pts indolent B-cell lymphoma 38 pts CLL 10 pts	-	DLBCL: 39% (18%) indolent B-cell lymphoma: 32% (12%) CLL: 0% (0%)	-	indolent B-cell lymphoma: 7.6 mos DLBCL (PR2D): 4 mos	-

*ADC* antibody–drug conjugate, *ALL* acute lymphocyte leukemia, *NHL* non-Hodgkin lymphoma, *CLL* chronic lymphocyte leukemia, *FL* follicular lymphoma, *pts* patients, *DLBCL* diffuse large B cell lymphoma, *ORR* overall response rate, *CR* complete response, *MTD* maximum tolerated dose, *PFS* progression-free survival, mos months, mDOR median duration of response, *EFS* event-free survival, *PR2D* recommended phase 2 dose, *OS* overall survival, *DL* dose level

Pinatuzumab vedotin, a novel anti-CD22 ADC, has rarely been used as a single agent in clinical settings. The efficacy of pinatuzumab vedotin with or without rituximab was tested in a phase 1 study of adult patients with diffuse large B-cell lymphoma (DLBCL), indolent B-cell NHL, and chronic lymphoblastic leukemia (CLL) [62]. At its recommended phase II dose, the overall response rate (ORR) was 36% in DLBCL and 50% in indolent B-cell NHL. Notably, no therapeutic effect was observed in CLL. The most common toxicity of pinatuzumab vedotin was peripheral neuropathy, especially peripheral sensory neuropathy.

## Improving the clinical efficacy of CD22 ADCs

An in vitro study [63] indicated that internalization ability influenced the cytotoxicity of inotuzumab ozogamicin. Clinical data showed that CD22 density on the tumor cell surface correlated with clinical outcomes [55, 61, 64]. *KMT2A* translocations/rearrangement is a high-risk cytogenetic factor closely associated with low CD22 expression, and positive minimal residual disease status after treatment with inotuzumab ozogamicin [23, 65]. The patients with higher baseline CD22 expression and normal cytogenetics benefited most from inotuzumab ozogamicin. Patients treated with inotuzumab ozogamicin also showed decreased CD22 expression in blasts at relapse [61, 64]. The mechanisms are unknown. Bryostatin-1 is a natural substance that can specifically elevate CD22 surface distribution in a dose- and time-dependent manner [66]. Bryostatin-1 upregulates CD22 expression on CLL cells by activating protein kinase C [66] and on B-ALL cells through potential membrane trafficking [67]. Consequently, bryostatin-1 can be used for pretreatment or in combination with CD22 ADCs [66], though clinical effects need exploration.

Unlike CD22 CAR-T cell therapy, most patients receiving CD22 ADCs are naive to CD19 CAR-T cell therapy. Inotuzumab ozogamicin can effectively reduce the tumor burden and usually serves as a bridging therapy to HSCT or CAR-T cell therapy. Many clinical trials are also exploring the combination of CD22 ADCs with rituximab or other chemotherapies to increase the response depth. Inotuzumab ozogamicin plus mini-hyper-CVD chemotherapy, with or without blinatumomab, represents a feasible therapeutic regimen for elderly patients with newly diagnosed Ph- B-ALL [68–70]. Inotuzumab ozogamicin plus rituximab, with or without other chemotherapy agents, has also elicited encouraging clinical results in treating R/R B-cell NHL [71–73].

## CD22 CAR-T cell structures and preclinical results

Unlike ADC, CAR consists of an extracellular antigen-recognizing single-chain variable fragment (scFv), a hinge and transmembrane domain, and an intracellular signaling domain. It can mimic the T-cells’ intrinsic activation mode and initiates their killing to tumor cells without the restriction of the major histocompatibility complex [74]. The investigation on CD22 CAR-T cell therapy is started with the scFvs from two recombinant immunotoxins, HA22 and BL22.

CAR-T cells using HA22-derived scFv did not exert enhanced cytolytic effects than that using BL22-derived scFv for the possible reason that HA22-derived scFv cannot produce a strong activation signal under sufficient antigen stimulation [75]. The novel fully human anti-CD22 antibody m971 has gradually become an appealing alternative to HA22. The epitope recognized by m971 is distinct from that by HA22 and BL22. HA22 and BL22 recognize IgG-like domain 3 of CD22, while m971 targets the most proximal three extracellular domains 5–7 with a relatively low avidity (Fig. 1A) [76]. Moreover, increasing the affinity of m971 scFv did not improve in vitro and in vivo CAR-T cell activity against CD22-low leukemia cells [67]. Second-generation CAR-T cells incorporating m971 scFv displayed a better anti-tumor activity than those with BL22 or HA22-derived scFv [37, 77]. This may be due to the decreased density of CD22 on the tumor cell surface caused by the HA22-mediated internalization, which was not observed with m971 antibody [76, 78]. The novel hCD22.7 scFv was shown to bind to the distal Ig-like domain 1 with high affinity without causing evident CAR-T cell-mediated antigen loss in a mouse model bearing the primary leukemia cells [79]. Several other CD22-targeted scFv structures have shown remarkable preclinical anti-tumor activity and have been adopted in clinical practice [21, 31]. Linkers between heavy and light chains [80] also affect the targeting capacity of CD22 CAR-T cells. Short linker facilitates the formation of immune synapse and spontaneous clustering of CARs without antigen stimulation, thus inducing a tonic activation and improved CAR-T cell function [81] (Fig. 1B).

## Efficacy and safety of CD22 CAR-T cell therapy

The available clinical results on CD22 CAR-T cell therapy [20, 21, 24–31] are summarized in Table 3. The first in-human phase 1 trial of CD22 CAR-T cells, conducted at the National Cancer Institute [20, 24], reported an ORR of 72% (n = 57) and CR rate of 70%. At a median follow-up of 2 years, median OS and relapse-free survival in CR patients were 13.4 months and 6.0 months, respectively; 35% of patients relapsed, and the majority developed CD22-dim or negative disease. Cytokine release syndrome (CRS) and neurotoxicity occurred in 72% and 86% of patients, respectively. Two pilot studies recruited three adult and five pediatric patients with B-ALL [81]. The ORR was 50% and all of the responders achieved CR, with the longest CR being 7 months. CRS occurred in 75% of patients, and one developed grade 3 CRS.

**Table 3:** Interim results of clinical trials of CD22 CAR-T cells

Clinical trial information	Institution	Transduction/costimulatory domain/scFv (CAR-T product or manufacture procedure)	Disease and patients	Prior CD19 CAR-T	CD19 negative or dim	Dosage	Pharmacokinetics	ORR(≥ CR, best response)	Prognosis	CRS at any grade (grade ≥ 3), evaluation criteria	Neurotoxicity at any grade (grade ≥ 3), evaluation criteria
Phase 1 NCT02315612 [20]	NCI	Lentivirus/4-1BB/m971	R/R B-ALL 21 pts	71.4%	47.6%	0.3 × 10^6^ cells/kg 1 × 10^6^ cells/kg 3 × 10^6^ cells/kg	Peak on D14, persist up to 18 mos	57% (57%)	-	76% (0%) Lee criteria	37.5% (0%) in first 16 patients
Phase 1 NCT02315612 [24]	NCI	Lentivirus/4-1BB/m971 (CD4/CD8 TCS)	R/R B-ALL 57 pts R/R DLBCL 1 pt	62%	56.9%	0.3 × 106 cells/kg 1 × 106 cells/kg 3 × 106 cells/kg	Peak on D14—D21, higher in those at CD4/CD8 TCS cohort	71.9% (70.2%) in evaluable 57 pts	mRFS (CR) 6.0 mos mOS (CR) 13.4 mos	86.2% (8.6%) Lee criteria	32.8% (1.7%) ASTCT criteria
Phase 1 ChiCTR-OIC-17013523 [21]	Beijing Boren Hospital	Lentivirus/4-1BB/- (YK-CD22BB-002)	R/R B-ALL 34 pts	91%	41.2%	0.2 ~ 34.7 × 10^5^ cells/kg	Peak on D12—D15 median persistence time was 28 days by FCM	81.3% (78.1%) in 32 evaluable pts	-	91.2% (2.9%) Lee criteria	17.6% (0%) CTCAE criteria
Phase 1 ChiCTR2000028793 [31]	Beijing Boren Hospital	Lentivirus/4-1BB/- (CD22-CARFH80)	R/R B-ALL 8 pediatric pts	100%	12.5%	0.68 ~ 9.4 × 10^6^ cells/kg	Peak on D11- D15	87.5% (75%)	-	87.5% (12.5%) ASTCT criteria	ICANS 25% (12.5%) ASTCT criteria
Two pilot studies NCT02650414 and NCT02588456 [81]	UPenn/Children’s Hospital of Philadelphia	Lentivirus/4-1BB/m971 (CART22)	R/R B-ALL 3 adult pts / 5 pediatric pts	25%	75%	39.6 ~ 500 × 10^6^ cells/pt	2 CR pts showed significant CAR-T expansion within D20	50% (50%)	-	75% (12.5%) Penn criteria	-
Phase 1 PLAT-07(NCT04571138) [25]	Seattle Children's Hospital	- /4-1BB/m971 (SCRI-CAR22v2)	R/R B-ALL 3 pts	100%	66.7%	2 × 10^5^ cells/kg	-	100% (100%)	-	-	-
New Treatment Measure Clinical Study ChiCTR1800019298 [26]	Tianjin First Central Hospital	-/4-1BB/-	R/R B-ALL 6 pts R/R DLBCL 7 pts	100%	33.3% (B-ALL)	DLBCL: 2.11 ± 0.24 × 10^6^ cells/kg B-ALL: 2.07 ± 0.42 × 10^6^ cells/kg	Peak on D14	DLBCL: 85.7% (57.1%) B-ALL: 33.3% (33.3%)	-	DLBCL: 42.9% (0%) B-ALL: 100% (16.7%) Lee criteria	ICANS 0% (0%) ASTCT criteria
Phase 1 NCT04150497(BALLI-01) [27]	Cellectis S.A	Lentivirus/4-1BB/- (UCAR-T22, disruption of TRAC and CD52 genes using TALEN technology)	R/R B-ALL 3 pts	33.3%	-	~ 1 × 10^6^ cells/kg	Peak on D9—D14	66.7% (33.3%)	-	33.3% (0%)	0% (0%)
Phase 1 NCT04088890 [28]	Stanford University School of Medicine	Lentivirus/4-1BB/m971 (CD4/CD8 T selection)	R/R LBCL 3 pts	100%	66.7%	1 × 10^6^ cells/kg	Peak on D14, persist up to 3 mos by qPCR	100% (100%)	-	100% (0%) ASTCT criteria	ICANS 0% (0%) ASTCT criteria
Phase 1 NCT04088890 (cohort expansion) [29]	Stanford University School of Medicine	Lentivirus/4-1BB/m971	R/R LBCL 21 pts	95%	-	1 × 10^6^ cells/kg 3 × 10^6^ cells/kg	Peak on D14	85.7% (66.7%)	mPFS not reached mOS not reached	100% (4.8%) ASTCT criteria	ICANS 19% (0%) ASTCT criteria
Phase 1 NCT02650414 [30]	UPenn	Lentivirus/4-1BB/m971 (CART22-65 s)	R/R B-ALL 17 pts	94.1%	100.0%	0.8 ~ 10 × 10^6^ cells/kg (3—day fractionated dosing)	Peak on D20	76.5% (76.5%)	mRFS 5.3 mos mEFS 5.8 mos mOS 16.5 mos	88.2% (0%)	35.3% (0%)

*NCI* National Cancer Institute, *UPenn* University of Pennsylvania, *TCS* T-cell selection, *UCAR-T* universal chimeric antigen receptor T-cell, *TRAC* T- cell receptor alpha constant, *TALEN* transcription activator-like effector nuclease, *R/R* refractory or relapsed, *ALL* acute lymphocyte leukemia, *LBCL* large B cell lymphoma, *FCM* flow cytometry, *ORR* overall response rate, *CR* complete response, mos months, *qPCR* quantitative real-time polymerase chain reaction, *mPFS* median progression-free survival, *mRFS* median relapse-free suvival, *mEFS* median event-free survival, mo months, *mOS* median overall survival, *NE* not evaluated, *CRS* cytokine release syndrome, *ASTCT* American Society for Transplantation and Cellular Therapy, *CTCAE* Common Terminology Criteria for Adverse Events, *ICANS* immune effector cell-associated neurotoxicity syndrome

We conducted a subgroup analysis based on the available clinical results of CD22 CAR-T cell therapy (Table 4**)**. While the pooled ORR did not differ with age, the CR rate was higher in children than that in adults (74% vs 57%, *P* = 0.05). Neurotoxicity tended to occur more frequently in children than adults (28% vs 11%, *P* = 0.04). In addition, CRS had a predilection for patients with B-ALL instead of those with B-cell lymphoma (87% vs 74%,*P* < 0.01). The relapse rate was higher in patients with B-ALL than that in patients with B-cell lymphoma (31% vs 8%, *P* = 0.04). Young patients also had a higher risk of relapse than adult patients (36% vs 6%,* P* = 0.01). Moreover, previous treatment with CD19 CAR-T cells did not influence the efficacy or safety of CD22 CAR-T cell therapy (CR 73% vs 79%, *P* = 0.63; CRS 76% vs 83%, *P* = 0.59; Neurotoxicity 13% vs 9%, *P* = 0.72).

**Table 4:** Subgroup analysis and exploration of heterogeneity in CD22 CAR-T clinical trials

Patients with available data	Overall response rate	Complete response rate	negative Minimal residual disease	Cytokine release syndrome	≥ Grade 3 cytokine release syndrome	Neurotoxicity	≥ Grade 3 neurotoxicity	Relapse rate	CD22 dim/negative relapse rate
Age Group									
Children (N = 114)	76% (68–83)	74% (65–81)	-	87% (80–92)	6% (3–12)	28% (21–37)	-	36% (18–54)	16% (0–32)
Adult (N = 37)	75% (59–87)	57% (41–72)	-	84% (68–93)	5% (1–19)	11% (4–25)	-	6% (0–20)	3% (0–9)
p value	0.94	0.05*	-	0.6	0.9	0.04*	-	0.01**	0.14
Bone marrow involvement (B-ALL)									
High burden (N = 98)	74% (65–82)	63% (43–83)	66% (55–75)	86% (78–92)	7% (3–14)	25% (17–34)	1% (0–7)	23% (6–58)	8% (1–46)
Low burden (N = 25)	81% (66–96)	76% (59–93)	68% (48–83)	88% (69–96)	4% (1–24)	32% (17–52)	4% (1–24)	40% (23–60)	12% (4–31)
p value	0.22	0.54	0.82	0.81	0.6	0.46	0.32	0.37	0.73
Disease									
B-ALL (N = 133)	76% (69–83)	72% (65–80)	-	87% (82–93)	4% (1–8)	20% (8–32)	1% (0–3)	31% (13–49)	11% (0–23)
B cell lymphoma (N = 28)	86% (73–99)	64% (47–82)	-	74% (19–100)	4% (0–12)	10% (0–28)	0% (0–6)	8% (0–19)	4% (0–12)
p value	0.19	0.41	-	< 0.01**	0.87	0.36	0.75	0.04*	0.35
Prior CD19 CAR-T therapy									
Yes (N = 67)	75% (65–87)	73% (62–86)	45% (16–74)	76% (62–91)	9% (3–30)	0% (0–6)	0% (0–6)	19% (0–46)	24% (9–66)
No (N = 15)	76% (58–100)	79% (58–100)	62% (22–100)	83% (62–100)	13% (3–58)	0% (0–6)	0% (0–6)	29% (5–52)	20% (7–60)
p value	0.76	0.64	0.5	0.59	0.72	1	1	0.59	0.55

Data are event rate, % (95% CI), p values, or number of patients. Adults were patients aged 20 years or older; children were patients aged younger than 20 years, *CAR* chimeric antigen receptor

## Overcoming treatment failure of CD22 CAR-T cells

Lower CD22 density in blasts and a higher tumor burden are associated with poor outcomes. Zheng et al. [82] reported two CD22 splicing isoforms—a Δex5-6-splicing and Δex2-skipping isoform—from the RNA sequencing databases of newly diagnosed pediatric B-ALL patients. The former isoform causes epitope recognition failure in the IgG-like domain 3, while the latter is a CD22 Δex2 variant (Δex2 encodes mRNA containing the initiation codon AUG) that cannot be translated into any identifiable protein. The CD22 variants may partially explain the initial low CD22 density on tumor cell surface. In addition, prior CD22-targeted immunotherapy is a potential predictor of poor efficacy. Tumors can escape the killing of CAR-T cells by decreasing the antigen density on the surface, which has been observed in both CD19 and CD22 CAR-T cell therapy. Notably, Ramakrishna et al. [67] found that the genetic sequence and mRNA levels of CD22 remained unchanged in the tumor cells of relapsed patients, but the surface distribution of CD22 decreased. Moreover, CD22 CAR-T cells did not induce obvious trogocytosis of CD22 on tumor cells [83]. Membrane trafficking resulted in the internalization of CD22 molecules after antigen–antibody interactions [78], which might explain the downregulation of CD22 after CAR-T cell therapy. Notably, CD22 splicing isoforms were not detected after treatment failure with CD22 CAR-T cells.

CD22 downregulation is an important mechanism of treatment failure after CD22 CAR-T cell therapy [20, 21, 29–31, 81]. Bryostatin-1 can sensitize tumor cells to CD22 CAR-T cell therapy [67]. However, bryostatin-1 cannot affect CD22 expression on tumor cell lines with low primary CD22 expression. Direct exposure to ﻿bryostatin-1 can dampen interferon-γ production while enhancing granzyme B secretion in CD22 CAR-T cells. In a mouse model, bryostatin-1 effectively prolonged in vivo persistence and promoted the memory phenotype of CD22 CAR-T cells [67]. Epigenetic modifiers, such as 5-azacytidine and all-trans retinoic acid can also modulate CD22 expression in different cell lines [84]. Although these studies provide proof for combination therapies with CD22 CAR-T cells, further research is needed to determine the optimal timing and duration.

Dual-targeting effectively mitigates the antigen loss associated with CAR-T cell therapy and improves treatment outcomes. Many dual-targeting strategies have been reported, including sequential infusion [85–88] and co-administration [89] of two CAR-T cell products, co-transduction [90, 91] or sequential transduction [92] of T cells with two vectors conveying different CARs, and transduction of T cells with one vector encoding two separate CARs (bicistronic structure) [93, 94], using tandem [95–101] or loop [102, 103] scFv structures. Preclinical studies demonstrated that tandem CD19/CD22 CAR-T cells eradicated CD19 + tumor cells effectively but had a limited cytotoxicity on CD22 + tumor cellss compared to single-target CAR-T cells [104]. However, tandem CD19/CD22 CAR-T cell therapy resulted in a higher CR rate than single-target or sequential infusion of CD19 and CD22 CAR-T cells [105, 106]. Recently, Kokalaki et al. [107] screened out 9A8 as a better scFv in CAR design based on its sensitivity to CD22-low tumors after sequential antigen stimulation. They constructed two CAR vectors incorporating the FMC63 or 9AB scFv and developed a dual-target CAR-T cell product using a co-transduction method. CD19/CD22 dual-target CAR-T cell therapy showed relative better efficacy than single-target CAR-T cell therapy, with a CR rate ranging from 83%–100% in B-ALL and 50%–62.5% in B-cell lymphoma [85, 86, 90–101] (Table 5). Trispecific CAR-T cells targeting CD19, CD20 and CD22 also showed a promising ability for eliminating antigen-heterogeneous tumor cells [108].

**Table 5:** Results of clinical trials of CD19/CD22 dual-targeting CAR-T cells

Clinical trial information	Institution	Dual-targeting strategy	CAR structures	Disease and patients	Prior CD19 CAR-T treatment at baseline	ORR(≥ CR, best response)	Prognosis	CRS at any grade (grade ≥ 3), evaluation criteria	Neurotoxicity at any grade (grade ≥ 3), evaluation criteria
Observational study ChiCTR-OPN-16008526 [86]	Tongji Hospital, Tongji Medical College, Huazhong University of Science and Technology	Sequential infusion without interval (D0-D3)	anti-CD19 scFv (Murine)/4-1BB anti-CD22 scFv (Murine)/4-1BB	R/R B-ALL 51 pts R/R B-cell NHL 38 pts	-	B-ALL: 98% (96%) in 50 evaluable pts B-cell NHL: 72.2% (50%) in 36 evaluable pts	B-ALL: mPFS 13.6 mos, mOS 31 mos B-cell NHL: mPFS 9.9 mos, mOS 18 mos	95.5%(17.9%) Lee criteria	CRES 13.5% (1.1%) CTCTE criteria
Observational study ChiCTR-ONC-17013648 [87]	Beijing Boren Hospital	Sequential infusion	FMC63 scFv/4-1BB anti-CD22 scFv (human)/4-1BB	R/R B-ALL 21 pts	100% (16 CR, 3 PR, 2 relapsed)	95% (95%)	18-month OS rate 88.5% 18-month EFS rate 67.5%	52% (0%) Penn criteria	0% (0%) CTCTE criteria
Phase 1 ChiCTR-OIB-17013670 [88]	Beijing Boren Hospital	Sequential infusion	anti-CD19 scFv/4-1BB anti-CD22 scFv/4-1BB	R/R B-ALL 20 pts	-	100% (100%)	mLFS/mOS not reached 1-year LFS rate 79.5% 1-year OS rate 92.3%	CD19 CAR-T 90% (5%) CD22 CAR-T 75% (0%)	CD19 CAR-T 15% (5%) CD22 CAR-T 15% (0%)
Phase 2 ChiCTR2000032211 [89]	Multicenter	Coadministration (1:1)	anti-CD19 scFv/4-1BB anti-CD22 scFv/4-1BB	B-ALL 6 pts R/R B-ALL 188 pts B-ALL with isolated EMD 31 pts	-	99% (99%)	12-month EFS rate 73.5%	88% (28.4%) ASTCT criteria	20.9% (4.0%) ASTCT criteria
Phase 1 NCT03289455 [93]	Autolus PLC	Bicistronic CAR-T	FMC63 scFv/OX40 LT22 scFv-COMP/4-1BB	R/R B-ALL 15 pts	-	86.7% ( 86.7%)	-	80% (0%) Lee criteria	ICANS 26.7% (0%) ASTCT criteria
Phase 1 (UCAR-T) NCT04227015 [95]	The First Affiliated Hospital, School of Medicine, Zhejiang University	Tantem CAR-T	FMC63 scFv-m971 scFv/4-1BB	R/R B-ALL 6 pts	-	83.3% (83.3%)	-	100% (16.7%)	0% (0%)
Phase 1 ChiCTR1800015575 [96]	The First Affiliated Hospital, School of Medicine, Zhejiang University	Tantem CAR-T	FMC63 scFv-anti-CD22 scFv(human)/4-1BB	R/R B-cell lymphoma 16 pts	-	87.5% (62.5%)	2-year OS rate 77.3% 2-year PFS rate 40.2% mPFS 246 days	100% (6.3%) ASTCT criteria	0% (0%) CTCAE criteria
Phase 1 NCT03185494 [97]	Institute of Basic Medicine, Chinese PLA General Hospital	Tantem CAR-T	m971 scFv-FMC63 scFv/4-1BB	R/R B-ALL 6 pts	-	100% (100%)	-	100% (0%) Lee criteria	ICANS 0% (0%) ASTCT criteria
Phase 1 NCT03233854 [102]	Stanford University School of Medicine	Loop CAR-T	FMC63 VH-m971 VL-m971 VH-FMC63 VL/4-1BB	R/R B-ALL 17 pts R/R LBCL 21 pts	DLBCL 65%	B-ALL: 100% (80%) LBCL: 62% (29%)	-	76% (5%) Lee criteria	37% (10.5%) CTCAE criteria
Phase 1 NCT03919526 [103]	Shanghai General Hospital, Shanghai Jiaotong University School of Medicine	Loop CAR-T	FMC63 VL-m971 VH-m971 VL-FMC63 VH/4-1BB	B-ALL 15 pts	-	100% (100%)	mRFS/mOS not reached 12-month RFS rate 77% 12-month OS rate 86%	26.7% (0%) ASTCT criteria	ICANS 0% (0%) ASTCT criteria

*PLA* Liberation Army General, UCAR-T universal chimeric antigen receptor T-cell, *R/R* refractory or relapsed, *ALL* acute leukemia lymphocyte, *CR* complete response, pt patient, *NHL* non-Hodgkin lymphoma, *PR* partial remission, *ORR* overall response rate, *mPFS* median progression-free survival, *mOS* median overall survival, *EFS* median event-free survival, mos months, *mLFS* median leukemia-free survival, *mRFS* median relapse-free suvival, mos months, *CRS* cytokine release syndrome, *ICANS* immune effector cell-associated neurotoxicity syndrome, *ASTCT* American Society for Transplantation and Cellular Therapy, *CTCAE* Common Terminology Criteria for Adverse Events

## Conclusion and future perspective

CD22 is a potential target especially for patients who have experienced treatment failure with CD19-targeted immunotherapies. CD22-targeted immunotherapies have shown promising efficacy and acceptable toxicities in hematologic malignancies. Despite the difference in patient characteristics, CD22 CAR-T cells outperformed CD22 ADCs in terms of clinical efficacy, reflecting the superiority of T-cell-mediated immunity over the cytotoxic agents. We also found that prior exposure to CD19 CAR-T cells did not profoundly affect the efficacy of CD22 CAR-T cells. Therefore, CD22 CAR-T cell therapy could either be an upfront choice if CD22 density is far higher than CD19 on the tumor cell surface or be a candidate for CD19 CAR-T cell therapy. CRS and neurotoxicity are typical toxicities caused by CAR-T cells. CRS could sometimes be intense and leads to multi-organ damage. But CD22 ADC provides a safer option for those at high risk to develop CAR-T-associated toxicities and the elders, at least as a bridging therapy. CD22 ADCs are not suitable for patients who relapsed after CD22 CAR-T treatment due to the associated antigen loss. Instead, CD22 ADCs can prepare the patients for subsequent CD19 CAR-T cell therapy, as the counts of CD3 + T cells are maintained after the treatment [61]. However, the prolonged B-cell aplasia caused by CD22 ADCs may be an unfavorable factor for subsequent CAR-T cell therapy [109], given that a lower percentage of CD19 + B cells (< 15%) in the bone marrow at infusion correlated with a shorter persistence of CD19 CAR-T cells [11]. One pediatric patient with B-ALL experienced a myeloid lineage switch after the infusion of CD19 CAR-T cells following the treatment with inotuzumab ozogamicin. Notably, *KMT2A* rearrangement was previously detected in the case, emphasizing the importance of detecting genetic abnormalities at enrollment [61].

Both CD22 ADCs and CAR-T cells were more effective in treating B-ALL than B-cell NHL. This was especially evident with CD22 ADCs, demonstrating a more suitable therapeutic profile against B-ALL. Therefore, both of CD22 ADC and CAR-T cell therapy are feasible for patients with B-ALL. But CD22 ADCs are not recommended to patients with DLBCL or large B-cell lymphoma. As for indolent lymphoma, limited data is accessible on CD22 CAR-T cell therapy, while CD22 ADCs  demonstrate modest efficacy compared to other targeted therapies [110, 111].

Nevertheless, relapse after CD22-targeted immunotherapies remains an unsolved problem. Particularly, the antigen downregulation is apparent in CD22-targeted therapies, and the mechanism is unclear. The preclinical results [66, 67, 83, 84] of bryostatin-1 plus CD22-targeted therapies uphold the combination therapies, but how to schedule and administrate the use of bryoststin-1 still needs more clinical exploration. CD19/CD22 dual-targeting CAR-T cells demonstrated excellent efficacy and can avoid antigen escape to a great extent. Based on the different mechanisms of immunotherapies, CD22 ADC plus CD22 CAR-T, CD19 CAR-T, or CD19 bispecific T cell engager provide new directions for designing effective therapeutic strategies. However, whether this would produce a result greater than one plus one remains unexplored.

Collectively, optimization of CD22 CAR-T cells and ADCs, combination strategies and dual-targeting designs are future perspectives, which shed light on the better clinical application of CD22-targeted therapies.

## Data Availability

All data generated or analyzed during this study are included in this published article.

## Abbreviations

ADC: Antibody–drug conjugate
B-ALL: B-cell acute lymphoblastic leukemia
CAR: Chimeric antigen receptor
CLL: Chronic lymphoblastic leukemia
CR: Complete response
CRS: Cytokine release syndrome
DLBCL: Diffuse large B-cell lymphoma
HSCT: Hematopoietic stem cell transplantation
NHL: Non-Hodgkin lymphoma
ORR: Overall response rate
OS: Overall survival
R/R: Relapsed or refractory
scFv: Single-chain fragment variable
SOS: Sinusoidal obstruction syndrome
VOD: Veno-occlusive disease
